# The Efficiency of Utilisation of High-strength Steel in Tubular Profiles

**DOI:** 10.3390/ma13051193

**Published:** 2020-03-06

**Authors:** Ieva Misiūnaitė, Viktor Gribniak, Arvydas Rimkus, Ronaldas Jakubovskis

**Affiliations:** Laboratory of Innovative Building Structures, Vilnius Gediminas Technical University, Sauletekio av. 11, LT-10223 Vilnius, Lithuania; Ieva.Misiunaite@vgtu.lt (I.M.); Arvydas.Rimkus@vgtu.lt (A.R.); Ronaldas.Jakubovskis@vgtu.lt (R.J.)

**Keywords:** cold-formed profiles, high-strength steel, local deformations, bending test, load-bearing capacity

## Abstract

The use of high-strength steel (HSS) is a current trend of the construction industry. Tubular profiles are widely used in various structural applications because of their high stiffness-to-weight ratio, exceptional resistance to torsion, and aesthetic appearance. However, the increase of the strength for the same elastic modulus of the material and geometry of tubular profiles is often not proportional to the rise of the load-bearing capacity of the structural element. The obtained experimental results support the above inference. The study was based on the flexural test results of two groups of HSS and normal-strength steel (NSS) tubular specimens with a 100 × 100 × 4 mm (height × width × thickness) cross-section. Numerical (finite element) simulation results demonstrated that the shape of the cross-section influenced the efficiency of utilisation of HSS. The relationship between the relative increase of the load-bearing capacity of the beam specimen and the corresponding change of the steel strength determined the utilisation efficiency.

## 1. Introduction

The use of advanced materials such as high-strength steel (HSS) [1,2,3,4,5,6] is a current trend of the construction industry. The increase of the strength of the materials enables the reduction of the self-weight of the structural elements. Tubular profiles are widely used in various structural applications because of their high stiffness-to-weight ratio, exceptional resistance to torsion, and aesthetic appearance. Hot-rolling and cold-forming processes are predominant in the production of the steels. The application result of the latter technology—a square hollow section (SHS) profile—is the research object of this study.

Production technology has a substantial effect on altering mechanical properties of the steel [7]. Characteristics of the hot-rolled material remain practically unchanged. However, the cold-forming process causes a strength enhancement of the steel, smoothing the shape of the resultant stress-strain diagram; at the same time, the process reduces ultimate deformations, increasing the brittleness of the steel [8,9,10,11]. Substantial plastic deformations appear in the corner regions of SHS that causes uneven distribution of material properties in the section [8,11].

The failure of tubular profiles is a consequence of the excessively increased deformations (the stiffness condition) or fracture of the steel (the strength term). The efficient utilisation of the material requires an equilibrium between the above limitations. On the other hand, an increase of the strength under the same elastic modulus and geometry of the profile causes inefficient utilisation of the material. Misiūnaitė et al. [6] revealed that local effects cause the failure of the SHS specimens made from both normal-strength steel (NSS) and HSS. However, the increase of the load-bearing capacity of the tubular profiles was not proportional to the rise of the strength of the steel. Several studies investigated the adequacy of slenderness limits, identifying the extent to which the local buckling controls the resistance of the section [12,13,14]. An alternative approach is based on the identification of a correlation between the cross-section resistance and deformation capacity [15]. However, none of the works mentioned above define conditions ensuring efficient utilisation of HSS in tubular profiles that can be nominally estimated as the ratio between the alteration of the load-bearing capacity of the element and the increase of the steel strength.

The effect of an increase of the material strength on the enhancement of the mechanical resistance of the profiles is the focus of this research. Two groups of HSS and NSS tubular beam specimens with 100 × 100 × 4 mm (height × width × thickness) cross-section were tested until failure, estimating the efficiency mentioned above. The experimental technique [6] was used to assess the local bearing capacity of the specimens. Numerical simulations illustrated the proposed efficiency concept.

## 2. Test Program

Square tubular profiles are not susceptible to torsional deformations. The stiff closed shape of the cross-section makes the elements resistant to lateral buckling. These aspects emphasise the structural efficiency of such profiles. At the same time, strengthening of tubular profiles, using supplemental materials, is a more complex problem than that which is characteristic of the open cross-sections [16]. Thus, the efficiency of the utilisation of HSS, reflecting the increase of the load-carrying capacity as the interaction between the local bearing mechanism and global bending resistance of the cold-formed tubular profiles, is the research object of this study.

### 2.1. Characterisation of the Materials

Flat coupons, extracted from the square hollow section (SHS) profiles, were used to determine material properties. Three tensile specimens were produced for each profile. One coupon was cut from the flange opposite to the welding seam; the other two coupons were extracted from the web. The 100 mm gauge length of the coupons had the thickness of the profile and the 20 mm width. The geometry corresponded to the standard EN ISO 6892-1 requirements [17]. This study employed two steel grades with the stress values of 355 MPa (NSS) and 900 MPa (HSS). Table 1 shows the chemical composition of the steel specified in the mill certificates.

**Figure 1:**
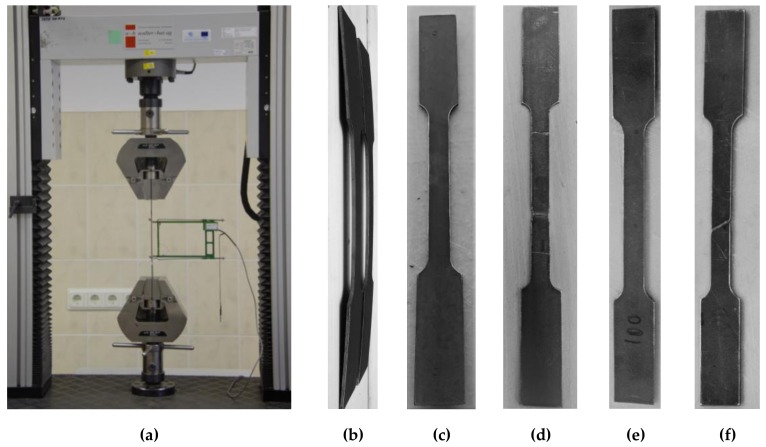
Tensile tests of the steel coupons: (**a**) test setup; (**b**) a deformed shape normal-strength steel (NSS) coupons cut from a straight square hollow section (SHS) profile; (**c**,**d**) NSS coupon before and after testing, respectively; (**e**,**f**) HSS coupon before and after testing, respectively.

**Figure 2:**
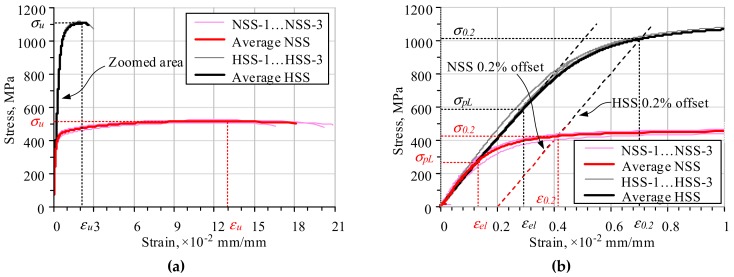
Stress–strain relations of flat tensile coupons of NSS and HSS: (**a**) actual graphs; (**b**) initial part of the diagrams

The universal electromechanical testing machine LFM 100 (walter + bay ag, Löhningen, Switzerland) of 100 kN capacity with system controller and data acquisition software Dion Stat (v.7, w + b Software, Löhningen, Switzerland) (the displacement control error was less than 0.1%) was utilised for the tension test (Figure 1a). Figure 1b shows the deformed shape of the coupons extracted from a nominally straight profile. Figure 1c–f demonstrate the selected coupon samples before and after testing. Figure 2 shows the stress–strain relationships of both considered steel grades. It also includes the graphs of each coupon (NSS-1…NSS-3 and HSS-1…HSS-3) and the averaged diagrams, which give the mean stresses corresponding to particular strain value. The outputs of the tension tests (Figure 1a) were collected every 0.05 seconds. Thus, more than 2000 records were composed in the stress-strain database. This made it possible to use the linear interpolation to harmonise the measurements of different test samples (coupons).

The averaged diagrams (Figure 2b) had a rounded shape with a moderate strain hardening that reflected typical material behaviour of carbon steel after the cold-forming process. Figure 2 demonstrates that the coupons made from HSS had a substantially increased strength, but decreased ductility comparing to the NSS coupons. The diagrams of both steel grades defined almost identical elastic moduli, which predominantly characterised the deformational behaviour of the profiles.

An adequate assessment of the elastic modulus is vital for materials having a smoothed stress-strain diagram (e.g., Figure 2) because it determines the equivalent yield stress (σ_0.2_) defining the theoretical strength of the material. The existing specifications [18,19,20] recommend two methods to estimate the elastic modulus. The approach [20] was based on the linear approximation of two characteristic points of the stress–strain diagram expressing the elastic modulus as

(1) $$E=\frac{\sigma_{2}-\sigma_{1}}{\varepsilon_{2}-\varepsilon_{1}}$$

where stresses *σ*_1_ and *σ*_2_ correspond to the strains *ε*_1_ = 0.0005 and *ε*_2_ = 0.0025, respectively. Such a simplifying assumption increases the sensitivity of the resultant estimation to non-linear effects caused by production peculiarities of the profiles. The cold-forming process induces residual stresses in the material, of which relaxation distorts the planar shape of the tensile coupons. Figure 1b shows the initial deformed shape of the coupons cut from a straight NSS profile. The stretching of such samples (coupons) caused a discrepancy of the “elastic” part of the stress–strain diagram from a linear form. Therefore, Equation (1) was not used to approximate the graphs shown in Figure 2b.

Another method to determine the elastic modulus employs a regression technique to describe the linear part of the stress-strain curve [17,18,19]—the slope of the regression line is the analysis parameter. No strict requirements exist to determine the limits of the linear portion of the stress–strain curve except the ISO [17] that defines the boundaries for the cold-formed carbon steel as 10% and 50% of the nominal value of the stress σ_0.2_.

In this study, the limits of the linear portion of the stress–strain relationship were determined by maximising two parameters: the determination coefficient value (that should be not less than 0.99) and the length of the analysed part of the diagram. Figure 2b shows the defined regression lines. Table 2 gives the corresponding material parameters. In the table, *E* is the elastic modulus; *σ*_0.2_ is the equivalent yielding stress*; σ_u_* is the ultimate stress; *ε_el_* and *ε*_0.2_ are the strains corresponding to the proportional stress *σ_pL_* and the equivalent yielding stress *σ*_0.2_, respectively (Figure 2b); and *A_gt_* is the elongation percentage at the maximum force. The elastic moduli of NSS and HSS were respectively estimated at the 30%–60% and 10%–50% ranges of the nominal stresses *σ*_0.2_. The assessment of the HSS agreed well to the range recommended by ISO [17], whereas the elastic modulus determination range of NSS was modified due to the initial non-linearity of the shape of the coupon samples (Figure 1b). The lower boundary of the elastic modulus range was arbitrarily increased up to 30% to minimise the straightening effect of the coupon.

### 2.2. Beam Tests

The previous investigation [6] revealed that local effects could cause the failure of the SHS specimens. The same procedure, based on the application of a digital image correlation (DIC) system, was utilised for monitoring the local deformations in this study. The four-point bending layout was used to induce a uniform distribution of stresses in the pure bending zone localising the ultimate strains close to the load application point. Linear variable displacement transducers (LVDT, Novotechnik, Southborough, Mass) monitored vertical displacements. The specimens were tested until failure.

**Figure 3:**
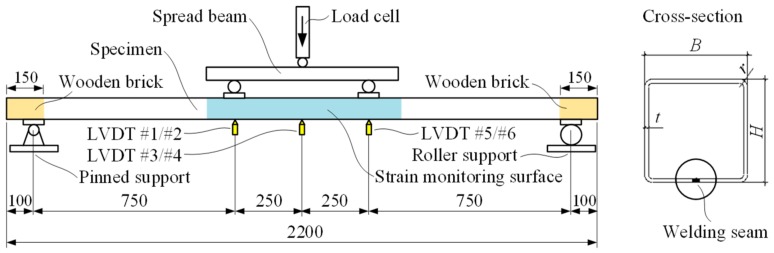
Loading scheme and cross-section of the beam specimen.

The experiments were carried out in two groups comprising HSS and NSS beam specimens; each group consisted of two tubular beams. Table 3 describes the geometry parameters of the flexural specimens. The notation letter of the specimens designates the normal-strength (“*N*”) or high-strength (“*H*”) steel, whereas the numeral corresponds to the specimen number. In Table 3, *B* and *H* are the width and height of the cross-section, *t* is the thickness of the profile, and *r* is the outer radius of the cross-section corner (the cross-section in Figure 3 defines the notations). The theoretical section moduli (elastic *W_el_* and plastic *W_pl_*) are also given in the table.

Figure 3 shows the loading scheme and the dimensions of the specimens. Steel rollers and bearing steel plates were used, ensuring the simple support conditions. Wooden bricks were put inside the profile to avoid the local failure at the supports. The loading points of the beams were left unstiffened to investigate the interaction of the local and global failure mechanisms of the profiles. The beams were loaded under displacement control using a servohydraulic testing machine of 5000 kN capacity; the loading rate was 0.1 mm/min. A load cell measured the reaction to the applied load.

As shown in Figure 3, paired LVDT were used to monitor vertical displacements in the pure bending zone—two 100 mm LVDT devices (#3 and #4) were located at the mid-span and two sets of the 50 mm LVDT (#1, #2, and #5, #6) were placed at the boundaries of the bending zone. A data logger Almemo 2890-9 recorded the reading of the load cell and all LVDT devices every second.

The DIC system monitored deformation of the surface (coloured in Figure 3) at the rate of one frame per kN. The monitored surfaces of the specimens were prepared (white painting with contrast pattern) to facilitate tracking the displacement tensors. The DaVis 8.1.6 software package by La Vision (Göttingen, Germany) was used to process the collected data. This system enables the monitoring of movements of any points arbitrarily set on the monitored surface after the physical tests [6].

## 3. Test Results

**Figure 4:**
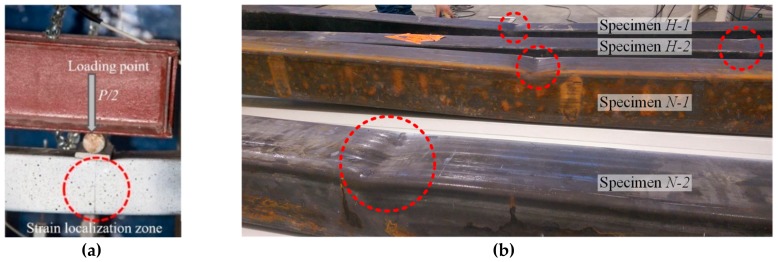
Failure localisation of the beams: (**a**) a typical failure; (**b**) the tested specimens.

As expected, the failure of all beam specimens was a consequence of the strain localisation close to the load application point (Figure 4a). Figure 4b demonstrates deformed shapes of the tested beams. The LVDT devices were unable to identify the local deformation mechanism. Therefore, these devices were used to assess the curvature of the pure bending zone and the global deformation of the profiles expressed in terms of the vertical displacements.

**Figure 5:**
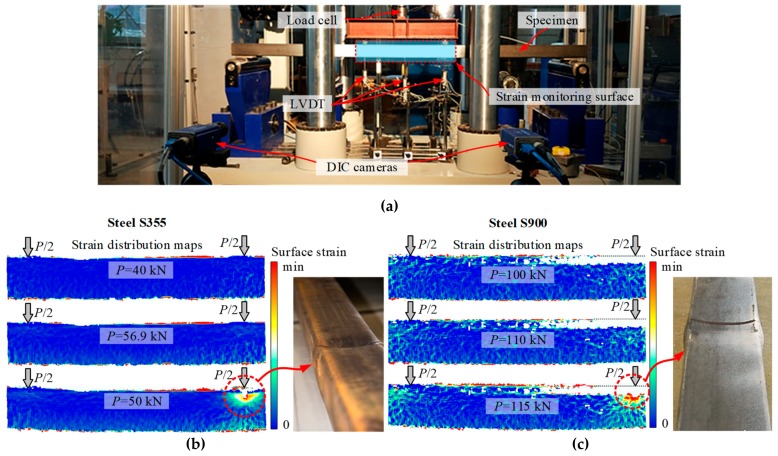
Deformation analysis of the SHS bending profiles: (**a**) loading sheme; (**b**,**c**) strain distribution maps identified by the digital image correlation (DIC) system and local failure of the NSS and HSS beam specimens.

The local deformation behaviour of the beam specimens was the focus of this study. The DIC system was used to monitor the strain localisation process. Figure 5a shows the stereoscopic layout of digital cameras. Figure 5b,c demonstrate the typical test results of NSS and HSS profiles.

**Figure 6:**
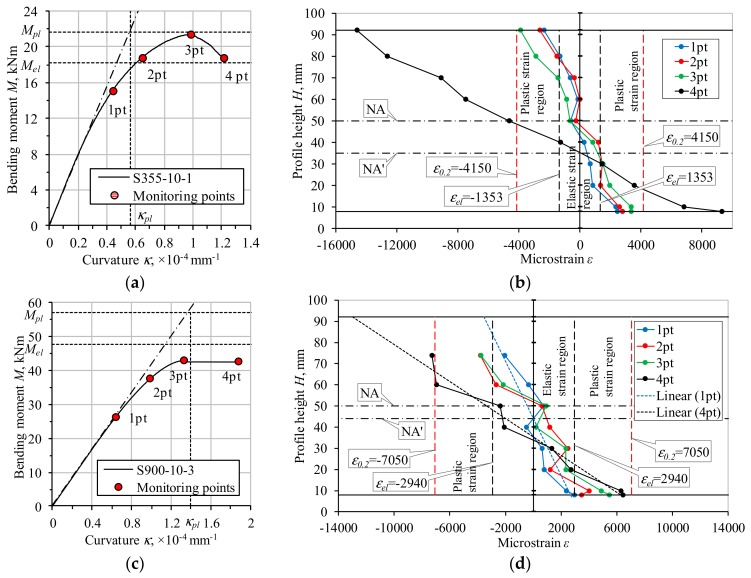
Moment-curvature diagrams: (**a**) NSS, (**b**) HSS; and strain distribution: (**c**) NSS, (**d**) HSS.

Although the deformed shape of the NSS and HSS profiles shown in Figure 4 and Figure 5b,c look similar, the corresponding deformation mechanisms were different. The respective compressive strain distribution maps (Figure 5b,c) identified by the DIC system illustrated the evolution of local deformations with the load increase. By comparing these maps, it can be observed that the strain localisation in the NSS profile corresponded to the descending branch of the loading diagram. Figure 6a shows the respective moment-curvature diagram. On the contrary, the HSS element demonstrated continuously increased deformations until the ultimate loading stage (see Figure 6b for reference).

Table 4 summarises the test results, where *M_u_* is the ultimate bending moment corresponding to the maximum load, and *M_el_* and *M_pl_* are the theoretical values of the elastic and plastic moments, respectively. Both theoretical bending moments were calculated using the yield strength *σ*_0.2_ from Table 2 and the corresponding section modulus from Table 3. The following equation determines the curvature of the pure bending zone $\kappa_{u}$, corresponding to the maximum bending moment *M_u_*:

(2) $$\kappa_{u}=\frac{24\delta_{L/2}}{3L^{2}-4L_{s}^{2}}$$

In Equation (2), $\delta_{L/2}$ is the vertical displacement at the mid-span defined as the average outcome of the LVDT devices #3 and #4 (Figure 3), and *L* and *L_s_* are the total span and the shear span of the beam, respectively (*L* = 2 m, *L_s_* = 0.75 m).

In parallel to the results shown in Figure 5b,c, Table 4 illustrates the difference between the deformation mechanisms, which are characteristic of the NSS and HSS profiles subjected to flexure. In this table, *M_u_* and *κ_u_* are the experimental ultimate bending moment and the corresponding curvature of the pure bending zone, and *M_el_* and *M_pl_* are the theoretical elastic and plastic moments, respectively. The actual bending resistance of the NSS specimens described by the moment *M_u_* is comparable to the theoretical plastic moment *M_pl_*—the tubular specimens were capable of resisting the equivalent yielding stress (*σ*_0.2_). In other words, the strength term controlled the load-bearing capacity of the NSS beams. Alternatively, the bending resistance of the HSS profiles was well below the theoretical moment *M_el_*. This means that the stiffness condition governed the failure behaviour of the HSS specimens.

**Figure 7:**
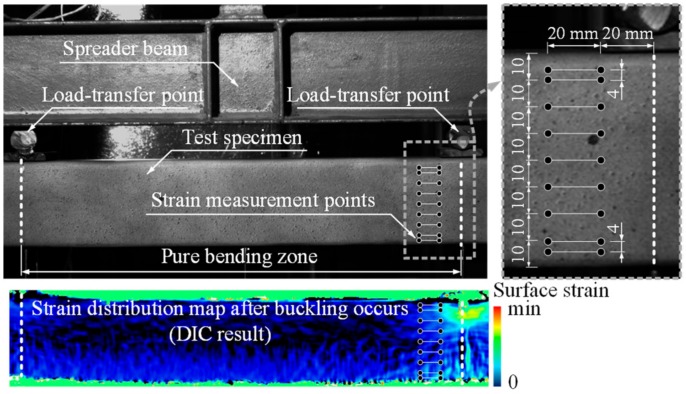
Virtual strain gauges of the DIC system.

A detailed analysis of the strain distribution maps generated by the DIC system (Figure 5b,c) was carried out to determine the failure mechanisms of the beam specimens. The virtual strain gauges created by the DaVis 8.1.6 software were applied to monitor the deformation localisation process near the loading point. Figure 7 shows the distribution of the 20 mm virtual gauges. Figure 6c,d show the deformation diagrams of the flat part of the web identified using the virtual devices. The grey-coloured areas define the elastic strain regions limited by the average value of the strain *ε_el_* given in Table 2. The red-filled zones correspond to the plastic strain regions limited by the average strain *ε*_0.2_ (Table 2). The strain distribution diagrams correspond to the loading points highlighted in the moment-curvature graphs shown in Figure 6a,b.

Figure 6c shows the strain distribution of the beam specimen *N-1*. It can be observed that the compressive strain, corresponding to the first point (“1pt”) of the diagram given in Figure 6a, exceeded the proportional limit *ε_el_*. The deformations did not transcend the yield limit *ε*_0.2_ until the load reached the third point (“3pt”). The shift of NA to the position NA’ at that moment caused the increase of the local deformations, but the specimen *N-1* was still capable of sustaining partial yielding before the failure occurred.

Although DIC system can help to monitor the failure mechanisms, even this technology is not always capable of capturing local deformations. Two HSS beam specimens were tested (Table 4), but only the specimen *H-1* demonstrated the evident deformation localisation process. There are two possible explanations for this outcome: (1) only one surface was exposed to the DIC, or (2) the deformation localisation process was uniformly distributed between the loading points.

Further analysis is based on the DIC results of the specimen *H-1*. Figure 6d shows the respective strain distribution. Unfortunately, the results of the virtual gauges placed in the compressive zone were lost due to an accidental light reflection. Therefore, linear trends were used to extrapolate the strain diagrams. The shift of NA to the position NA’ indicated a premature nonlinear deformation of the HSS bending profile. This process can be observed at the loading level corresponding to the first point (“1pt”) of the diagram shown in Figure 6b. It also demonstrated that the deformations were localised in the outer layers of the section up to the third load point (“3pt”). The failure of the beam specimen was a consequence of the strain concentration realised before the yielding limit that reveals a predominant role of the local buckling effects on the deformation behaviour of the HSS profile.

## 4. Discussion of the Results

The relationship between the enhancement of the resistance of the SHS profiles to the mechanical load and the increase of the strength of the material is the focus of this research. The test results showed that the increase of the material strength for the same elastic modulus and geometry of SHS was not proportional to the rise of the load-bearing capacity of the tubular element subjected to bending. Comparative analysis of the local deformational behaviour of the SHS profiles identified the differences of failure mechanisms of the NSS and HSS specimens

- NSS: The beam specimens were capable of attaining the load-bearing resistance comparable to the theoretical plastic moment *M_pl_*. The considered geometry of SHS enabled the efficient utilisation of the material. The *M_u_*/*M_el_* ratio given in Table 4 defined the average increase of the theoretical load-bearing capacity by 18%.
- HSS: A premature local buckling limited the load-bearing capacity of the tubular specimen. The considered geometry and structural performance of the profile disabled the ability to utilise HSS efficiently due to local bearing failure. The *M_u_*/*M_el_* ratio (Table 4) demonstrated the 9% deficit of the ultimate load, considering the theoretical elastic moment as the reference.

An appropriate alteration of the cross-section geometry is necessary to ensure efficient structural utilisation of advanced materials. The existing design codes employ a slenderness limit approach to identify the extent to which the local buckling resistance controls the load-bearing capacity of a tubular profile. The equivalent slenderness of the flange or web of the cross-section can be calculated using Equation (3), as follows:

(3) $$\lambda =\frac{b}{t}\sqrt{\frac{f_{y}}{E}}$$

where *b* is the width of the flat part of the web or flange, *t* is the thickness of the corresponding part of the cross-section, *f_y_* is the yield strength of steel, and *E* is the elastic modulus.

Several investigations [12,13,14] were carried out to verify the applicability of the slenderness limit for the structural design using HSS tubular profiles. Misiūnaitė et al. [6] proved the adequacy of this approach for the analysis of HSS profiles considered in this study. Equation (3) indicates that the slenderness increases together with the strength (*f_y_*) if all other input parameters are remaining constant. Such estimation agrees well with the results of this study.

**Figure 8:**
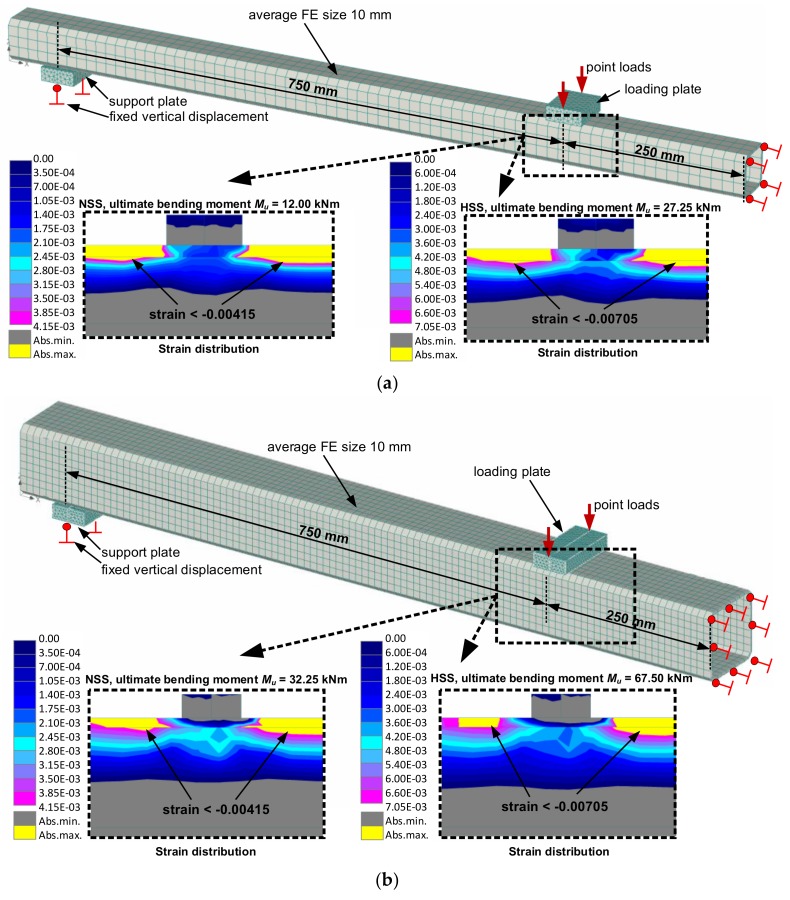
Finite element (FE) model and numerical simulation results (strain distribution) of the beams with different size of the cross-section: (**a**) 60 × 60 × 4 mm; (**b**) 100 × 100 × 4 mm.

A finite element (FE) model developed and verified in the referred work [6] is used to illustrate the efficiency concept. For simplicity, the four-point bending test with the symmetry condition at the mid-section of the profile is the simulation object. The loading scheme is the same as it was used in the physical experiment (Figure 3). Figure 8 shows the FE models and boundary conditions. Two cross-sections are modelled. One SHS (100 × 100 × 4 mm) is the same as was used for the physical tests (Section 2.2), whereas another profile (60 × 60 × 4 mm) is considered as the alternative for demonstrating the utilisation efficiency of HSS. The FE size (= 10 mm) is identical in both models.

The relationship between the relative increase of the load-bearing capacity of the beam specimen $\Delta M_{u,HSS/NSS}$ and the corresponding ratio of the steel strengths (grades) $\Delta \sigma_{0.2,HSS/NSS}$ determines the HSS utilisation efficiency. The following coefficient is used for that purpose:

(4) $$\begin{array}{ccc} c_{ef}=\frac{\Delta M_{u,HSS/NSS}}{\Delta \sigma_{0.2,HSS/NSS}}; & \Delta M_{u,HSS/NSS}=\frac{M_{u,HSS}}{M_{u,NSS}}; & \Delta \sigma_{0.2,HSS/NSS}=\frac{\sigma_{0.2,HSS}}{\sigma_{0.2,NSS}} \end{array}$$

where *M_u,HSS_* and *M_u,NSS_* are the maximum bending moments, and *σ*_0.2*,HSS*_ and *σ*_0.2*,NSS*_ are the equivalent yielding stresses. The subscripts HSS and NSS correspond to the normal-strength steel and high-strength steel, respectively.

The manufacturing process of SHS profiles affects the mechanical properties of steel, inducing residual stresses in the corner regions [11] and enhancing the yield strength [7,8,9,10,11]. These mechanisms compensate each other and can be neglected in numerical simulations [11,21]. Thus, the FE analysis employs a simplified material modelling concept—the von Mises material model with bilinear stress-strain diagram and strain hardening was used. The experimental stress–strain relationships (Figure 2b) determined the material properties of the NSS and HSS profiles. The possible geometry imperfections were also neglected following the recommendation by the referred work [21]. The simulations were performed incrementally increasing the load (with 1 kN increment) until failure of the beam. The equivalent strain limit *ε*_0.2_ was chosen as the failure criterion. As it can be observed in Table 2, the strain limits of 4.15‰ and 7.05‰ correspond to NSS and HSS profiles, respectively. Figure 8 shows the simulation results. For the 100 × 100 × 4 mm cross-section, the estimated ultimate bending moment increased from 32.3 to 67.5 kNm (2.09 times) when NSS was replaced with HSS. For the alternative cross-section (60 × 60 × 4 mm), the corresponding increases reached 2.28 times (from 12.0 to 27.3 kNm). The comparison of the equivalent stresses *σ*_0.2_, presented in Table 2, gave the ratio of 2.36 (= 1013/430). The coefficient *c_ef_* (Equation 4) defined the 89% utilisation of HSS in the 100 × 100 × 4 mm profile, whereas the 96% effectiveness was possessed in the 60 × 60 × 4 mm cross-section. In other words, the latter profile enables the utilisation of the HSS in a more efficient way than the specimen used for the physical tests.

The actual efficiency of the utilisation of HSS in the 100 × 100 × 4 mm profile, however, is less significant than that was observed in the physical tests. On average, the coefficient *c_ef_* was equal to 85% for the data presented in Table 4. This is a consequence of the assumption of the simplified material models in the FE analysis; the deformation interaction mechanism between the flange and web was ignored as well. Thus, the identification of the optimum configuration of the cross-section is an object for further research.

## 5. Conclusions

Although the current trends in the construction industry are related to the use of high-strength steel (HSS), the increase of the load-bearing capacity of the member is often not proportional to the rise of the strength of the material. This study employed the flexural test results of two groups of HSS and normal-strength steel (NSS) tubular specimens with 100 × 100 × 4 mm cross-section to illustrate this observation. A simplified finite element (FE) model was used to demonstrate the efficiency assessment principles. The ratio *c_ef_* between the relative increase of the load-bearing capacity of the structural member and the respective increment of the steel strength was the parameter for defining the utilisation efficiency. The obtained results enabled the formulation of the following conclusions:

- An almost identical initial elastic modulus was characteristic of the considered steel grades S355 (NSS) and S900 (HSS). This parameter predominantly controlled the deformation behaviour of the elements.
- The failure of all beam specimens was a consequence of the deformation localisation process identified by using a digital image correlation system. Although the deformed shape of the profiles looked similar, the corresponding deformation mechanisms were different. The strain localisation in the NSS profile corresponded to the descending branch of the loading diagram, whereas the HSS element continuously demonstrated increased deformations until the ultimate loading stage.
- The actual bending resistance of the NSS profiles was comparable to the theoretical plastic bending moment *M_pl_*. This meant that the strength term controlled the load-bearing capacity of the NSS elements. The *M_u_*/*M_el_* ratio gave the average increase of the theoretical load-bearing capacity at 18%. Alternatively, the stiffness condition governed the failure behaviour of the HSS specimens; the load-bearing capacity was below the theoretical value *M_el_* at 9%. The identification of the optimum configuration of the cross-section should be the subject for further research.
- The numerical analysis demonstrated that the HSS utilisation efficiency was dependent on the shape of the cross-section. The ratio *c_ef_* was found as being equal to 89% for the 100 × 100 × 4 mm profile, whereas it increased up to 96% for the 60 × 60 × 4 mm cross-section. The actual efficiency of the utilisation of HSS in the 100 × 100 × 4 mm specimen was less substantial than that obtained in the physical tests (*c_ef_* = 85%). This was a consequence of the idealised approximation of the deformation localisation process in the tubular profiles assumed in FE analysis.

## Figures and Tables

**Table 1:** Chemical composition of the steel (%).

Material	C	Si	Mn	P	S	Al	Cr	Ni	Mo	Cu	Nb	V	Ti
HSS	0.100	0.250	1.30	0.020	0.010	0.015	–	–	–	–	0.050	0.050	0.070
NSS	0.140	0.180	1.13	0.006	0.014	0.030	0.040	0.020	–	0.040	0.020	–	0.003

**Table 2:** Averaged mechanical properties of the steel.

Steel Grade	*E* (GPa)	*σ*_0.2_ (MPa)	*σ_u_* (MPa)	*ε_el_* (×10^−3^)	*ε*_0.2_ (×10^−3^)	*σ_u_*/*σ*_0.2_ (–)	*A_gt_* (%)
S900 (HSS)	200.6	1013	1112	2.94	7.05	1.1	2.07
S355 (NSS)	200.0	430	515	1.35	4.15	1.2	13.03

**Table 3:** Geometry parameters of the beam specimens.

Specimen	*B*	*H*	*t*	*r*	*W_el_*	*W_pl_*
	(mm)	(mm)	(mm)	(mm)	(×10^3^ mm^3^)	(×10^3^ mm^3^)
*N-1*	100.33	100.43	3.75	7.49	43.26	51.51
*N-2*	100.62	99.80	3.72	7.45	42.70	51.22
*H-1*	101.74	101.60	3.99	7.98	46.81	56.33
*H-2*	101.65	101.68	4.00	8.00	46.93	56.53

**Table 4:** Results of the bending tests.

Specimen	*M_u_*	*κ_u_* × 10^−4^	*M_el_*	*M_pl_*	*M_u_*/*M_el_*	*M_u_*/*M_pl_*
	(kNm)	(mm^-1^)	(kNm)	(kNm)	(–)	(–)
*N-1*	21.34	1.00	18.17	21.63	1.17	0.99
*N-2*	21.41	1.01	17.93	21.38	1.19	1.00
*H-1*	43.05	1.35	47.44	57.08	0.91	0.75
*H-2*	43.13	1.26	47.56	57.29	0.91	0.75
